# Health Professionals' Knowledge of how to Report a Missing Person With Dementia: A National Survey

**DOI:** 10.1093/geroni/igaa057.204

**Published:** 2020-12-16

**Authors:** Margaret MacAndrew, Elizabeth Beattie, Dubhglas Taylor, Jim Whitehead, John Quinn

**Affiliations:** 1 Queensland University of Technology, Camp Hill, Queensland, Australia; 2 Queensland University of Technology, Brisbane, Australia; 3 Brisbane, Australia; 4 Queensland Police, Brisbane, Australia

## Abstract

In Australia one in five land searches conducted by Police involve a person with dementia. Over a third of these people go missing from a health care service and 15% are not found alive. Delays in commencing a specialised search for the missing person with dementia contributes to the risk of death. Delays in Police searching may result from ambiguity in current policies about how to report a missing patient/client. This study aimed to explore health professional’s knowledge about how to report a missing person with dementia and reasons for delayed reports to Police. 246 Australian health professionals completed an online survey. Most were registered nurses (n=124), allied health professionals (n=69) and medical practitioners (n=22) who worked in a range of settings including acute care (n=111), community care (n=59) and residential aged care (n=44). Over a third (n=81) did not know their care service policy for reporting a missing patient/client and did not know if their health service had a policy specific to reporting a missing person with dementia. 20% did not know how long they needed to wait before reporting a missing person to Police and fear of calling Police too soon or wasting their time were common reasons for delaying a report. These findings confirm a degree of misunderstanding about current policy and procedures for reporting a missing person with dementia. Addressing knowledge deficits and standardising approaches to reporting a missing person with dementia in Australia would be recommended as a step toward improving their health outcomes.

